# Supplementary material for: Prevalence of Methicillin and β−Lactamase Resistant Pathogens Associated with Oral and Periodontal Disease of Children in Mymensingh, Bangladesh
Source: Pathogens. 2022 Aug 9;11(8):890. doi: 10.3390/pathogens11080890 (PMC9414569; doi:10.3390/pathogens11080890)
Supplement: Supplementary file 1 [file pathogens-11-00890-s001.zip › Supplemental table S1.pdf]

Supplementary Table S1. List of primers used in this study to detect bacteria causing OPD, its virulence genes and antibiotic resistance genes

**Target genes**		**Primer sequence (5’−3’)**	**Amplicon size (bp)**	**References**
S. aureus	*nuc*	F-GCGATTGATGGTGATACGGTT<br>R-AGCCAAGCCTTGACGAACTAAGC	276	Mehmood *et al.,* (2020)
S. salivarius	*gtfK*	F-GTGTTGCCACATCTTCACTCGCTTCGG<br>R-CGTTGATGTGCTTGAAAGGGCACCATT	544	Hoshino *et al.*, (2004)
S. mutants	*gtfb*	F –ACTACACTTTCGGGTGGCTTGG<br>R-CAGTATAAGCGCCAGTTTCATC	517	Oho *et al.*, (2000)
S. sobrinus	*gtfi*	F-GATAACTACCTGACAGCTGACT<br>R-AAGCTGCCTTAAGGTAATCACT	712	Oho *et al.*, (2000)
L. fermentum	*lf*	F-AATACCGCATTACAACTTTG<br>R-GGTTAAATACCGTCAACGTA	337	Dickson *et al.,* (2005)
Virulence gene	*ace*	F-GGAATGACCGAGAACGATGGC<br>R-GCTTGATGTTGGCCTGCTTCCG	616	Salah *et al.,* (2011)
	cylA	F-GACTCGGGGATTGATAGGC<br>R-GCTGCTAAAGCTGCGCTTAC	688	Salah *et al.*, (2011)
	efaA	F-GCCAATTGGGACAGACCCTC<br>R-CGCCTTCTGTTCCTTCTTTGGC	688	Creti*et al.,* (2004)
	gelE	F-CGAAGTTGGAAAAGGAGGC<br>R-GGTGAAGAAGTTACTCTGA	372	Al-Talib *et al.*, (2015)
	hyl	F-ACAGAAGAGCTGCAGGAAATG<br>R-GACTGACGTCCAAGTTTCCAA	276	Vankerckhoven*e t al.,* (2004)
	clfA	F-CCGGATCCGTAGCTGCAGATGCACC<br>R-GCTCTAGATCACTCATCAGGTTGTTCAGG	1000	Arciola CR *et al.*, (2002)
Antibiotic resistance genes	*nim*	F-ATGTTCAGAGAAATGGGGCGTAAG<br>R-GCTTCCTTGCCTGTCATGTGCTC	458	Alauzet *et al.*, (2010)
	blaTEM	F-AGATCAGTTGGGTGCACGAG<br>R-CAGTGCTGCAATGATACCGC	618	Alsultan *et al.*, (2013)
	cfxA	F-GCGCAAATCCTCCTTTAACAA<br>R-ACCGCCACACCAATTTCG	802	Sedgley *et al.,* (2008)
	mecA	F-AAAATCGATGGTAAAGGTTGG<br>R-AGTTCTGGCACTACCGGATTTTGC	533	Lee *et al.*, (2003)
