# Supplementary material for: Prevalence of Methicillin and β−Lactamase Resistant Pathogens Associated with Oral and Periodontal Disease of Children in Mymensingh, Bangladesh
Source: Pathogens. 2022 Aug 9;11(8):890. doi: 10.3390/pathogens11080890 (PMC9414569; doi:10.3390/pathogens11080890)
Supplement: Supplementary file 1 [file pathogens-11-00890-s001.zip › Supplemental table S2.pdf]

Supplementary Table S2. Accession numbers of the sequenced genes of the respective pathogens in the current study

**Target organism/gene**	**Sample ID**	**Sample type**	**Sample source**	**Strains**	**GenBank Accession No.**
S. aureus	BAU-OS35Sa-nuc	Homo sapiens	Oral swab	BAU-OS35	MZ067380
	BAU-OS45Sa-nuc	Homo sapiens	Oral swab	BAU-OS45	MZ067381
	BAU-OS47Sa-nuc	Homo sapiens	Oral swab	BAU-OS47	MZ067382
	BAU-OS52Sa-nuc	Homo sapiens	Oral swab	BAU-OS52	MZ067383
	BAU-OS60Sa-nuc	Homo sapiens	Oral swab	BAU-OS60	MZ067384
	BAU-OS62Sa-nuc	Homo sapiens	Oral swab	BAU-OS62	MZ067385
	BAU-OS64Sa-nuc	Homo sapiens	Oral swab	BAU-OS64	MZ067386
	BAU-OS65Sa-nuc	Homo sapiens	Oral swab	BAU-OS65	MZ067387
	BAU-OS66Sa-nuc	Homo sapiens	Oral swab	BAU-OS66	MZ067388
	BAU-OS96Sa-nuc	Homo sapiens	Oral swab	BAU-OS96	MZ067389
S. salivarius	BAU-OS8Ss-gtfk	Homo sapiens	Oral swab	BAU-OS8	MZ067328
	BAU-OS9Ss-gtfk	Homo sapiens	Oral swab	BAU-OS9	MZ067329
	BAU-OS16Ss-gtfk	Homo sapiens	Oral swab	BAU-OS16	MZ067330
	BAU-OS75Ss-gtfk	Homo sapiens	Oral swab	BAU-OS75	MZ067331
	BAU-OS77Ss-gtfk	Homo sapiens	Oral swab	BAU-OS77	MZ067332
	BAU-OS80Ss-gtfk	Homo sapiens	Oral swab	BAU-OS80	MZ067333
	BAU-OS83Ss-gtfk	Homo sapiens	Oral swab	BAU-OS83	MZ067334
	BAU-OS84Ss-gtfk	Homo sapiens	Oral swab	BAU-OS84	MZ067335
	BAU-OS95Ss-gtfk	Homo sapiens	Oral swab	BAU-OS95	MZ067336
	BAU-OS96Ss-gtfk	Homo sapiens	Oral swab	BAU-OS96	MZ067337
	BAU-OS98Ss-gtfk	Homo sapiens	Oral swab	BAU-OS98	MZ067338
L. fermentum	BAU-OS1LF-BD	Homo sapiens	Oral swab	BAU-OS10	MW940841

	BAU-OS2LF-BD	Homo sapiens	Oral swab	BAU-OS41	MW940842
	BAU-OS3LF-BD	Homo sapiens	Oral swab	BAU-OS74	MW940843
	BAU-OS4LF-BD	Homo sapiens	Oral swab	BAU-OS77	MW940844
	BAU-OS5LF-BD	Homo sapiens	Oral swab	BAU-OS86	MW940845
	BAU-OS6LF-BD	Homo sapiens	Oral swab	BAU-OS93	MW940846
	BAU-OS7LF-BD	Homo sapiens	Oral swab	BAU-OS94	MW940847
	BAU-OS8LF-BD	Homo sapiens	Oral swab	BAU-OS95	MW940848
	BAU-OS9LF-BD	Homo sapiens	Oral swab	BAU-OS96	MW940849
	BAU-OS10LF-BD	Homo sapiens	Oral swab	BAU-OS100	MW940850
clfA	BAU-OS45Sa clfA	Homo sapiens	Oral swab	BAU-OS45	MZ067242
	BAU-OS35Sa-clfA	Homo sapiens	Oral swab	BAU-OS35	MZ067243
	BAU-OS47Sa-clfA	Homo sapiens	Oral swab	BAU-OS47	MZ067244
	BAU-OS51Sa-clfA	Homo sapiens	Oral swab	BAU-OS51	MZ067245
	BAU-OS52Sa-clfA	Homo sapiens	Oral swab	BAU-OS52	MZ067246
	BAU-OS60Sa-clfA	Homo sapiens	Oral swab	BAU-OS60	MZ067247
	BAU-OS62Sa-clfA	Homo sapiens	Oral swab	BAU-OS62	MZ067248
	BAU-OS66Sa-clfA	Homo sapiens	Oral swab	BAU-OS66	MZ067249
	BAU-OS73Sa-clfA	Homo sapiens	Oral swab	BAU-OS73	MZ067250
	BAU-OS81Sa-clfA	Homo sapiens	Oral swab	BAU-OS81	MZ067251
mecA	BAU-OS13Sa-mecA	Homo sapiens	Oral swab	BAU-OS13	MZ067339
	BAU-OS35Sa-mecA	Homo sapiens	Oral swab	BAU-OS35	MZ067340
	BAU-OS62Sa-mecA	Homo sapiens	Oral swab	BAU-OS62	MZ067341
	BAU-OS64Sa-mecA	Homo sapiens	Oral swab	BAU-OS64	MZ067342

	BAU-OS66Sa-mecA	Homo sapiens	Oral swab	BAU-OS66	MZ067343
	BAU-OS96Sa-mecA	Homo sapiens	Oral swab	BAU-OS96	MZ067344
	BAU-OS52Sa-mecA	Homo sapiens	Oral swab	BAU-OS52	MZ067345
blaTEM	BAU-OS38Str-blaTEM	Homo sapiens	Oral swab	BAU-OS38Str	MZ067274
	BAU-OS75Ss-blaTEM	Homo sapiens	Oral swab	BAU-OS75Ss	MZ067275
	BAU-OS83Ss-blaTEM	Homo sapiens	Oral swab	BAU-OS83Ss	MZ067276
	BAU-OS81Lf-blaTEM	Homo sapiens	Oral swab	BAU-OS81Lf	MZ067277
	BAU-OS86Lf-blaTEM	Homo sapiens	Oral swab	BAU-OS86Lf	MZ067278
	BAU-OS90Lf-blaTEM	Homo sapiens	Oral swab	BAU-OS90Lf	MZ067279
	BAU-OS90Lf-blaTEM	Homo sapiens	Oral swab	BAU-OS90Lf	MZ067280
	BAU-OS129Lf-blaTEM	Homo sapiens	Oral swab	BAU-OS129Lf	MZ067281
